# “Distress is probably the wrong word”: exploring uncertainty and ambivalence in non-clinical voice-hearing and the psychosis continuum

**DOI:** 10.1080/17522439.2024.2407138

**Published:** 2024-09-25

**Authors:** Ariel Swyer, Angela Woods, Amanda Ellison, Ben Alderson-Day

**Affiliations:** a Department of Psychology, Durham University, Durham, UK; b Institute of Medical Humanities, Durham University, Durham, UK

**Keywords:** Voice-hearing, hallucinations, psychosis, distress, spirituality

## Abstract

**Background:**

Non-clinical voice-hearers (NCVHs) have been the subject of a growing body of psychological research, a primary aim of which is the development of new therapeutic techniques to support those who struggle with voice-hearing. However, relatively little research has examined non-clinical voice-hearing experiences beyond their relationship with clinical voice-hearing.

**Methods:**

The present study consists of a qualitative re-analysis of 17 semi-structured interviews conducted as part of an NCVH neuroimaging study which included items from the Psychotic Symptoms Rating Scale (PSYRATS) and Positive and Negative Syndrome Scale (PANSS). Results were generated using thematic analysis.

**Results:**

Analysis of interview responses showed that participants often experience negative voice content and negative emotion, but have frameworks which normalize a range of voice-hearing experiences. Participants also reported experiences which are not captured by standard clinical scales, as well as reporting comfort with uncertainty and ambiguity surrounding voices.

**Discussion:**

These results indicate that much of the experience of NCVHs may be missed by clinical measures and concepts, suggesting a need to approach them in ways that go beyond typical understandings of the psychosis continuum.

## Introduction

Central to the idea of a psychosis continuum is the existence of voice-hearing in the absence of a need for clinical care (Johns et al., 2014). Comparative studies of voice-hearing in clinical and non-clinical populations suggest that non-clinical voice-hearers (NCVH) report experiences that are broadly similar to those seen in the context of illness, often with no significant differences in loudness, location, or frequency of voices being observed (Daalman, Boks et al., 2011; Powers et al., 2017). Such similarities render NCVHs a useful comparison group for clinical voice-hearers (CVHs), and a body of research has sought to identify patterns of similarity and difference across clinical status. A central goal of such work is the development of therapeutic supports for those who struggle with voices. Two key areas of focus for such work are distress and appraisal.

Distress has emerged as a key factor which differentiates clinical and non-clinical voice-hearing, with a body of research having found that NCVHs experience little to no voice-related distress, or less voice-related distress than CVHs (Baumeister et al., 2017; Daalman, Boks et al., 2011; Hill et al., 2012). Daalman, Boks et al. (2011) for instance found that CVHs reported more overall distress than NCVHs and that emotional valence of voices was a major predictor of health status, reporting that the mean score for NCVHs on distress signalled “almost no discomfort, almost no disruption to daily life”. In research distress has often been measured as part of a wider assessment of hallucinations such as the Psychotic Symptoms Rating Scale (PSYRATS; Haddock et al., 1999), although some voice-hearing studies also include measures of anxiety and depression (Mawson et al., 2010). On the PSYRATS, distress refers to an individual’s emotional response to voices and is distinguished from negative voice-content (Haddock et al., 1999).

However, although a great deal of evidence supports the idea that voices are less distressing for NCVHs, the comparative emphasis of many studies has allowed little detailed examination of more ambivalent and negative feelings in non-clinical voice-hearing. Given that distress plays such an important role in voice-hearing literature and is often seen as a central–even definitional–difference between clinical and non-clinical populations, there is a need for a more nuanced understanding of distress and the ways in which distress may be experienced by NCVHs.

A related concept is appraisal, which refers broadly to the ways in which experiences are interpreted and the meaning attributed to them (Peters et al., 2017). Appraisal has also been implicated in voice-related distress and been shown to differ across clinical and non-clinical populations (Mawson et al., 2010). Such a difference is significant because it suggests that helping clinical voice-hearers to alter their voice appraisals could reduce distress and need for care.

Research on appraisal is rooted in a cognitive model of psychosis, which posits that beliefs about voice-hearing, as distinct from the content of the voices, have a crucial impact on clinical outcomes (Peters et al., 2017). Appraisal has been used in the literature to describe beliefs ranging from those about voice-characteristics, e.g. “malevolence” (Andrew et al., 2008), to possibilities for voice-interaction, e.g. “controllability”, to voice-source, e.g. “supernatural vs. biological” (Peters et al., 2017). Such research seeks to identify individual appraisals or combinations of appraisals which give rise to distress, as well as those which differ across clinical status. Evidence suggests that distress is correlated with perceived abilities and characteristics of voices, such as voice-omnipotence (Hacker et al., 2008), and whether the voice can be controlled (Hill et al., 2012). Compelling evidence for the role of appraisal in clinical status is offered by Peters et al. (2017) who found that non-clinical voice hearers appraised experimentally induced anomalous experiences differently from clinical voice-hearers, being less likely to find them threatening.

A principle aim of research on non-clinical voice-hearers, such as the work outlined above, is the development of new therapeutic interventions for clinical voice-hearers. This work, while important, tends to view non-clinical voice-hearers through the lens of illness, typically employing clinical research measures such as the PSYRATS (Haddock et al., 1999) and PANSS (Kay et al., 1987) which focus on concepts relevant to the treatment of clinical voice-hearers, such as clinical distress. Studies of non-clinical voice-hearing will often use such measures to establish the validity of their status (and therefore comparability to clinical groups) before going on to deploy cognitive and neuroimaging methods (Alderson-Day et al., 2017; Linden et al., 2011), but often only report summary scores rather than more detailed phenomenological descriptions of the underlying experiences. This kind of approach may overlook crucial points about non-clinical voice-hearing. For instance, voices which feel neither clearly internal nor external will not be captured by a question requiring a binary assessment of voices as internal or external. With few exceptions, the phenomenology of NCVH *per se* is rarely focused upon (c.f. Honig et al., 1998; Leudar et al., 1997).

The aim of the present study is to attempt a more nuanced account of the non-clinical voice-hearing experience, via a qualitative analysis of interview data gathered from a previous fMRI study with NCVH (Alderson-Day et al., 2017). The research included administration of the PSYRATS and PANSS interview schedules as part of a semi-structured interview on voice-hearing experiences. While symptom scores based on these interviews were reported at the time, the interviews gathered a range of in-depth descriptions of voice-experiences which warranted further examination. The present study sets out to do this through a thematic analysis of these interviews. Qualitative analysis of such data offers a unique opportunity not only to gain a nuanced account of the experiences of NCVHs but to see how well these experiences are captured (or not) via commonly used interview methods.

## Method

### Participants

Participants consisted of 17 non-clinical voice-hearers recruited via word-of-mouth, an online article in the Guardian newspaper, and spiritual communities from across the United Kingdom (see Alderson-Day et al., 2017 for full description of recruitment). Five participants who were excluded from the original neuroimaging study – due to being unable to complete the fMRI procedure – were nevertheless included in this study because they had full interview data available. Participants were between 18 and 68 years old, with 12 females and 5 males. The majority (13/17) of participants were White-British. Based on screening procedures in other NCVH studies (Sommer et al., 2010), participants had to be over the age of 18 and had to hear voices as defined by endorsement of at least one of the following items from the Launay-Slade Hallucination Scale: (a) “In the past I have had the experience of hearing a person’s voice that other people could not hear”, (b) “have heard a voice on at least one occasion in the past month”, (c)“I have been troubled by hearing voices in my head”. Participants were excluded if they had any psychiatric diagnosis other than anxiety or depression in remission (Alderson-Day et al., 2017), and none were help-seeking for their voices. Participants had a mean score of 4 on PANSS items relating to hallucination with SD = 0.60. On the PSYRATS, participants reported voice-frequency of constantly (2/17), every day (8/17), at least once a week (4/17), or once a month (3/17).

### Procedure

In Alderson-Day et al. (2017), NCVH participants were given semi-structured interviews made up of PSYRATS (Haddock et al., 1999) and PANSS (Kay et al., 1987) items relating to their experiences of and beliefs about voices in the past week. Additionally, they were encouraged to offer general descriptions of their voices at the beginning of the interview to contextualize their experiences. The interviews were recorded and transcribed by a professional transcriber, as part of the original study. At the time of recruitment, participants consented to analysis of their interview data in standalone research (i.e. separate from the eventual MRI study). In the present study, they were analysed by the lead author (AES). Names and identifying information were removed during transcription.

### Analysis

Reflexive thematic analysis (Braun & Clarke, 2022) was used to explore the main themes in the data. Reflexive thematic analysis is a flexible qualitative approach which aims to generate themes or “central organizing concepts”. These are distinct from “topics” (Braun & Clarke, 2019) and may be relevant to one or more specific areas of scientific interest such as distress and appraisal, but are not based on pre-determined categories. The analysis was inductive and took a semantic approach, striving to offer a rich account of several key themes, rather than choosing themes which offered an overarching picture of the data set (Braun & Clarke, 2006). As there is already a small body of work offering qualitative analysis of non-clinical voice-hearing (e.g. Roxburgh & Roe, 2014; Taylor & Murray, 2012), we took this approach to provoke new lines of investigation, by highlighting novel patterns (Braun & Clarke, 2006). After the data had been transcribed, AES read the data several times noting observations and familiarizing herself with the interviews. These notes were then used to generate initial coding frames which were discussed with the other authors (AW, BAD, and AE). Transcripts were then re-read and the codes formulated into initial themes. These were reviewed in relation to the transcripts and discussed again as a team. A final round of theme revision then followed with each theme reviewed to ensure they accurately reflected the data.

### Reflexivity statement

In reflexive thematic analysis, themes are ultimately generated by the researcher through an active process. It is therefore important to reflect on positionality and the ways in which this may interact with the analysis. The lead author (AES) does not hear voices and is therefore fundamentally describing and speaking for a group which she is not a member of. She has also worked in clinical settings with voice-hearers who had been diagnosed with hallucination-related conditions. This means that she has thought about voice-hearing as a symptom of clinical experience and spent time with those who have been supported by clinical approaches. This has also led her to spend time with individuals who oppose and have even been harmed by clinical approaches to voice-hearing. In this research, she has attempted to think about voice-hearing neither in line with nor defined in opposition to disorder, but as a phenomenon of its own. However, it is undeniable that her clinical experiences have influenced her.

## Results

Three superordinate themes were identified in the analysis: (1) “Beyond voices”, referring to voices which are not very voice-like and non-voice anomalous experiences, (2) “Distress is probably the wrong word”, which describes negative voice-experiences which are upsetting but not overwhelming and the normalization of negative content, and (3) “Holding uncertainty and ambiguity”, which describes participants’ ability to manage indeterminacy about the origin and nature of their experiences.

### Beyond voices

Many participants described experiences which were difficult to categorize both in terms of sensory modality and in terms of such qualities as internality and externality. At times, they could not pinpoint the sensory modality of an experience. Voices were experienced as “vibrations” or “electricity” or “like pouring water into a glass”.

#### I always felt something was responding to me: developing voices over time

While some participants reported that their first voices came on suddenly as distinctly voice-like, many reported that their experiences had been preceded by unusual experiences which were not voices or that the voices had become clearer over time. Several participants described a sense of communication which had “always” been present, slowly developing into actual voices.

I always felt something was responding to me, whether it was a voice or not I couldn’t really identify. (Harriet)

Yeah. So … they been … I’ve always been having this sense that I’m communicating with things. (Joan)

Others described voices as becoming clearer over time:

It was like quite incomprehensible voices in me, head, and then later on I sort of hacked them/sifted through them and distinguished a few. (Kyle)

these voices started to get stronger, as I sort of progressed in my thirties, even in my late twenties as well. (Patricia)

This slow and subtle onset, sometimes stretching back as far as participants could remember meant that many participants could not clearly identify their first experience of voice-hearing.

#### Resisting categorization

Participants often resisted defining their experiences as voices or expressed an inability to categorize them according to questions posed by PSYRATS. At times, some even resisted calling them voices:

I can’t differentiate whether I felt something or whether I heard something. (Harriet)

I wouldn’t be able to limit to a specific sensory input, like-voices, it’s just a general awareness, like getting … like a witnessing. (Oscar)

Joan reported that she hadn’t made a distinction between externality or internality or even conceptualized her experiences as voices until coming into contact with other voice-hearers.

I think when I was young I didn’t make that much of a distinction obviously. And I didn’t call it hearing voices, I don’t think I started calling it hearing voices till I met the Hearing Voices Network. (Joan)

Overall, voice-phenomenology- even in terms of whether or not they were voices – was often difficult to articulate and did not fit clearly into categories laid out by the interview measures.

### “Distress is probably the wrong word”

Distress and negative content were often described as present but not overwhelming. Ten out of seventeen participants described some form of negative voice-content, but this was generally attributed to a negative “entity” or the voice’s personality, rather than the experience of voice-hearing itself.

#### Normalization of negative experience

Participants who had spiritual frameworks for voices anticipated occasional negativity, due to the existence of negative spirits or energies.

If (the voices) do tell me off that’s a bad spirit coming through … that’s bad energy coming in, and I have had that experience as well and it’s not nice, so. (Harriet)

Other participants attributed negative experiences to a normal range of human behaviour on the part of voices:

I don’t think anyone’s ever been like aggressive or violent or … well of course I mean … I’ve channelled people who swear a lot but it’s like … it’s like God bless you to them, it’s like they’re not gonna be … it wasn’t directed at me, it was just these characters. (Ellie)

Along with normalizing voice-hearing, spiritual contexts gave some voice-hearers the power to cope with negative experiences Anna had a strong belief in God, who helped her cope with an unpleasant voice:

The closer and the more power that I received from God, the less he (voice) had. (Anna)

You get some people coming through who are very … forthright! However, you want to put it … Who are very forthright … then again it’s a case of you being in charge. (Mary)

There was also normalization of negative emotional experience itself. Anna explicitly rejected the word distress in describing her belief in the naturalness of a range of emotions:

Naturally when something is distressing, I allow myself to be somewhat … not distressed, upset about something. So distress is probably the wrong word, I will rarely get distressed, I let myself be upset about things that are naturally upsetting, that’s a normal human emotion, and to not do that would be … you know, would mean that I was numb to life, and I’m not you know, I cry at things that at sad and I cry at … you know I get emotional when I hear a beautiful piece of music. (Anna)

Belief systems which anticipated a range of voice-behaviour and, in some cases, gave participants power to take charge of voices, allowed participants to experience voice-related unpleasantness and distress without being overwhelmed by it. This allowed them to feel upset by individual experiences with voices, without being distressed by voice-hearing itself.

#### Distressing experiences surrounding voice-onset

Participants who reported voice-hearing experiences which were particularly upsetting described these as having taken place many years prior to the interview, surrounding the onset of voice-hearing. These participants reported significantly less voice-distress in the present day:

When I first started to experience it … because that’s how it was for a long time, and I was frightened, I was like a frightened person … and I … he (the voice) was shouting at me and it was very, very scary. (Anna)

(I) started having critical voices and voices talking about suicide and … ehm … and that was … that was partly as an accumulation of stress in my life. (Joan)

Joan links these early experiences with voices to “accumulated stress in her life” giving meaning to and normalizing the difficult period. Participants generally expressed an accepting attitude towards such early episodes of distressing voices:

I thought, right, well it has happened, I can’t do anything about it, and in actual fact, as they said afterwards, it hasn’t really spoilt your life, has it? You know you’ve still got your friends, you’ve still got your husband, your home, we haven’t really destroyed your life. I said, no, seven months of it maybe but not all of it. And I thought, well you’re going to have to let it go. (Daphne)

Participants described a variety of emotions relating to their voice-experiences, including unpleasant ones. However, frameworks and attitudes which normalized and accepted a range of emotion and voice-behaviour allowed them to cope with these experiences.

### Holding uncertainty and ambiguity

#### I can’t tell you where they’re coming from

While most participants had ideas about who or what the voices were – for example, spirits or parts of their own minds – many expressed some degree of uncertainty, not subscribing fully to a particular explanatory model or expressing openness to other models. The participants with the most certainty about the cause of voices, were participants who believed that the voices were spirits of the dead. However, many participants, including those who had a spiritual outlook, expressed some openness about voice-origin.

I just feel they can interact in this dimension, whoever “they” are, I don’t know. (Daphne)

Even some participants who did not express spiritual views and viewed voices as coming from their own minds occasionally expressed some uncertainty:

I have wondered in the past if I am picking up bits of other people’s conversations somehow, but it didn’t seem … you know the mechanism by which that might happen didn’t seem very likely, so I really don’t know. (Beth)

I may talk about this voice as another person, it just simplifies you know dealing with it and, thinking about it like that, although I’m completely open to other interpretations. (Claire)

There was an awareness among participants that there could be multiple explanations for the voices. One participant explicitly described her belief framework as *“the framework I’ve got at the moment!”* (Joan)

#### I don’t believe in good and evil

Along with seeming comfortable with uncertainty about the origins of voices, many participants seemed to accept voices as playing a complex role in their life and not being purely good or bad. There were exceptions to this, with some participants viewing voices as primarily pleasant or unpleasant, but even then, participants rarely invoked terminology beyond “nasty” to describe unpleasant ones.

This acceptance of ambiguity was sometimes apparent in participants with spiritual outlooks:

I don’t believe in concepts like good and evil … There are positive things and negative things, but … they’re discourse, they’re things people have made up, and I find that spiritual creatures don’t fall into good and evil, just things that want things and things that don’t want things. (Patricia)

It was also seen in participants who discussed voices as though they had human personalities:

He’s maybe said a couple of things a couple of times about obviously me being quite ugly and he’s been quite nasty about a couple of people in my family but that’s about it…Aye, he’s usually alright, as I said a few times he’s been quite snarky but you know on a sort of weekly basis he’s usually pretty docile. (Frances)

Participants’ acceptance of uncertainty about the origins of their voices, as well as their willingness to approach the voices and the world more broadly from a position of ambiguity and complexity, was a core part of how they experienced the emotional character of their experiences.

## Discussion

This research sought to gain a more nuanced picture of the ways in which key concepts in voice-hearing research – notably, distress and appraisal – operate in context for non-clinical voice-hearers. It highlighted the multifaceted nature of distress in NCVHs, with most participants reporting negative experiences with voices, but that these experiences were rarely overwhelming and often acceptable as a normal aspect of life. Furthermore, contextual factors such as strong sense of self and support systems interacted with the ways in which voices were appraised, perhaps making distressing experiences more manageable. Highlighting the complicated role of appraisal in voice-hearing, NCVHs in this study described uncertainty about voice origin and comfort with the ambiguity of the experience. Along with pointing to the complexity of previously established categories, this analysis identified areas that warrant further investigation, including the frequent report of “a sense of communication” preceding the onset of more fully formed “voice” experiences and other anomalous experiences which take place alongside voice-hearing.

Although not all participants reported distress or negative content, the fact that many did is of clinical relevance. These accounts are not consistent with the notion that non-clinical voice-hearing consists of mostly positive or neutral content (Daalman, van Zandvoort, et al., 2011). With distress having been identified as a variable which consistently differs across clinical and non-clinical populations (Baumeister et al., 2017) – even constituting a definitional difference in some cases – there is risk of painting inaccurate picture of the non-clinical voice-hearing experience as one which is free of difficulty and negative emotion. The participants in this study frequently described negative experiences, but these experiences did not overwhelm them or envelope their lives. Furthermore, negative experiences were anticipated by participants and normalized, as either part of the landscape of spiritual experience (you run into “negative energy”) or that of human personality and mood (voices can be “a bit snarky”).

Clinically based understandings of the word “distress” itself may lead research to overlook or de-emphasize the range of negative or otherwise complicated emotions that are part of the voice-hearing experience. This word was explicitly rejected by participants who nevertheless described negative emotional experiences with voices. Participants often spoke of voices that did not fit neatly into the categories of “positive” or “negative” or could be captured by a linear scale of related “distress”, instead offering descriptions that contained the complexity and multiplicity that comes with most human experience.

These descriptions also challenge conceptions of appraisal and belief framework as fixed and unambiguous. Participants often had a basic sense of their voices as either arising from their own minds or from spiritual sources, but notably, many participants expressed openness to both frameworks, often saying that they didn’t know for sure, or displaying an ability to hold both of these as possibilities. The notion that accepting uncertainty about voice origin may be beneficial has already been put forward by voice-hearers themselves: in a guide for navigating voice-hearing based on his own experiences and work with others, Dmitriy Gutkovich encourages voice-hearers to “label beliefs with ‘maybe’” (Gutkovich, 2020, p. 20).

In a study of agency in individuals with schizophrenia and psychosis (Jones et al., 2016), a participant reports that the pain of “in-betweenness” led her to want to be “unambiguously crazy”. Others in the same study report that an active process of interpretation and meaning-making, and a desire to make their experiences fit a particular model was involved in the development of psychosis. This stands in stark contrast to the ways in which participants in the present study seemed content to not fully understand their experiences, often reporting that they ultimately “did not know”, what they were or expressing openness to multiple explanations. Participants also displayed a willingness to hold ambiguity about the nature of the voices, often reporting that the same voice could be variously kind, cruel, “snarky”, “docile”, and “alright”. Although some participants described voices, particularly spirits, as “negative”, or “bad” many held more complicated views of voices and their intentions. This problematizes attempts to categorize non-clinical voice appraisals into such rigid categories as “benevolent”, and “malevolent” and raises the question of whether the firmness and complexity of one’s beliefs about voices may be as relevant to clinical status as the content of one’s beliefs.

Along with highlighting the role of uncertainty about voice origin, this work raised questions about how voices themselves are defined, and the broader context of voice-hearers’ phenomenological worlds. Participants described a range of anomalous experiences that fell outside of the bounds of “voice-hearing”, or hallucination in another modality, often preceding the onset of fully developed “voices”. Notably, several participants described early experiences of “communication” which they did not consider voices but connected with the later development of voices. This is in line with experiences described in *Psychosis Outside of the Box*, a project which has collected accounts of altered perceptual experiences which take place in the context of psychosis but are not captured by standard labels such as “voices” (Pagdon & Jones, 2020).

Of potential relevance to these descriptions is a model of schizophrenia which posits that “ipseity disturbance”, or a disturbance to one’s core self lies at the heart of the illness, accounting for all of the seemingly disparate symptom domains (Sass & Parnas, 2003). In this model, a progression of altered self-experience precedes the development of fully formed hallucination. Although it is premised on the idea of schizophrenia as a distinct object and is generally viewed as incompatible with continuum models of psychosis, aspects of ipseity disturbance model may be of relevance to NCVH research. Participants’ accounts of early “communication” and other nonvoice anomalous experiences suggest that non-clinical AVH may take place within a constellation of other altered experiences, perhaps bearing similarity to the way in which the ipseity disturbance model posits that those with schizophrenia experience “a profound, gestaltic transformation of the stream of consciousness”, which eventually results in hallucination (Raballo, 2016). In the ipseity disturbance model, this gestaltic transformation is conceptualized in terms of increasing psychological vulnerability and as a consciousness that is “disturbed”. What if, however, this idea of a transformation of consciousness were considered in a non-clinical context, as altered but not disturbed? A closer examination of non-hallucinatory anomalous experiences in NCVHs and the ways in which individuals respond to these experiences may be beneficial. In discussing an early sense of communication, several participants note that these had “always” been present, thus raising the question of whether some non-clinical voice-hearers have broader alterations to their experience of mind and the self, of which voice-hearing is just one part.

### Limitations and directions for future research

This study has several limitations which must be considered when interpreting the results. First, our analysis involved using secondary data from pre-existing interviews, which were structured to provide specific symptom ratings. This limited the extent to which the lead author could fully familiarise themselves with the data and the opportunity for follow-up points of ambiguity and complexity. Nevertheless, even within such limits, it was possible to identify a range of complex themes in the data; we are currently working with a tailored phenomenological interview to gather more extensive data in NCVH groups. Second, the sample for the study was not ethnically heterogeneous, and like other NCVH research, included many spiritual voice-hearers, limiting its representativeness. Future research on NCVHs should strive to address this, as it is a frequent limitation in NCVH research and may reflect underlying issues in recruitment strategy. Finally, these interviews were conducted at one point in participants’ lives and were not followed up later in time. Therefore, we do not know if any participants have, or will go on to, develop a voice-related disorder. Evidence from similar cohorts (e.g. Daalman et al., 2016) suggest only a minority of NCVH do so, but as many as 40% seek mental health support for other reasons. Our current research is building on such follow-ups to identify who, if any, have transitioned to psychosis.

## Conclusion

This research sought to gain a nuanced picture of the non-clinical voice-hearing experience, finding that, in many ways, participants’ descriptions complicated and resisted the concepts included in standard assessments of voice-hearing. Participants reported a mix of emotional experiences related to voices, including negative experiences which were not well captured by the concept of distress, as well as phenomenological experiences that defied categorization. Many were part of contexts and communities that normalized voice-hearing and these interacted with voice interpretation. Participants also conveyed a comfort with the uncertainty regarding the origin of their voices and ambiguity in regard to their nature. These results suggest a need to go beyond clinical measures and concepts and start asking new questions of voice-hearers of all clinical status. We suggest that, as researchers, tolerating some of the ambiguity and complexity of this phenomena will open up new avenues of study and offer a deeper understanding of the voice-hearing experience.

## Supplementary Material

Supplementary Material Interview Schedule.docx
