# Supplementary material for: “Distress is probably the wrong word”: exploring uncertainty and ambivalence in non-clinical voice-hearing and the psychosis continuum
Source: Psychosis. 2024 Sep 25;18(1):19–29. doi: 10.1080/17522439.2024.2407138 (PMC11616618; doi:10.1080/17522439.2024.2407138)
Supplement: Supplementary Material Interview Schedule.docx [file RPSY_A_2407138_SM4874.docx]

**Appendix A: PSYRATS/PANSS Combined Interview Schedule**

Use for assessing AVH primarily, along with other positive and negative symptoms. Section 1 covers voices; section 2 covers other behaviours and experiences. Include questions in grey for PANSS general assessment.

**SECTION 1**

**Preamble**

*For this interview I’m going to be asking some questions about the different kinds of experiences you’ve been having recently; and I’m mostly going to ask questions about things that have happened over the past week. Maybe we could start a bit more generally though. Could you describe to me some of experiences you’ve been having?*

**1. Frequency**

*How often have you heard voices over the past week?*

**2. Duration**

*When you have heard them, how long did they last?*

**3. Location**

*When you heard voices, where did it sound like they were coming from? (if that makes sense)*

**4. Loudness**

*The* ***last*** *time you heard a voice (even if it was longer ago than last week), how loud was it?*

**5 Beliefs about origin**

*What do you think has caused your voices? How convinced are you by that idea?*

**6. Amount of negative content**

*Have the voices said unpleasant/negative things over the past week?*

**7. Degree of negative content**

*What kind of things have the voices said over the past week?*

**8. Amount of distress**

*Have you found the voices distressing over the last week?*

[Cover no/under 10% distressing/under 50%/over 50%/always distressing]

**9. Intensity of distress**

*If the voices have been distressing over the past week, how intense was that?*

**10. Disruption to life**

*How much disruption to your life have the voices caused over the past week?*

**11. Controllability of voices**

*What control have you had over your voices over the past week? Can you get rid of them, or bring them on?*

**Optional items**

- **Number of voices?**
- **Form of each voice** (reference, 1^st^/2^nd^/3^rd^ person)
- **Sex of voices**

**SECTION 2**

*OK, now I’m going to ask about other kinds of experiences: how you’ve been feeling, what’s been on your mind, and so on.*

**2. Delusions/Thought Content**

*Have things been going well for you?*

*Has anything been bothering you recently?*

*Do you have any particular thoughts on life and its purpose? Do you follow a particular philosophy?*

*Do you believe in the Devil?*

*Can you read other people’s minds? How does that work?*

*Can others read your mind? How do they do that? Is there a reason they do?*

*Do you think anyone controls your thoughts?*

**3. Suspiciousness**

*How do you tend to spend your time?*

*Do you prefer to be alone?*

*Do you join in activities with others? If not, why not?*

*Do you have many friends?*

*Close friends?*

*Do you feel that you can trust most people? Are there any particular people you don’t trust?*

*Do you get along well with others?*

*Do you like most people? Do they like you?*

*Do you think other people talk behind your back? Do you feel like anyone is spying on you?*

*Do you sometimes feel in danger? Is someone thinking of harming you?*

**6. Grandiosity**

*How do you compare to the average person? Do you come out a little better, or a little worse? About the same?*

*Are you special in some ways?*

*Would you consider yourself gifted?*

*Do you have talents or abilities that most people don’t have?*

*Do you have special powers? Where do they come from?*

*Do you have ESP?*

*Are you very wealthy?*

*Do you consider yourself very bright?*

*Are you famous? Do people recognize your from the TV or radio?*

*Are you a religious person?*

*Are you close to God? Has God assigned you a special role or purpose?*

*Do you have a special mission in life?*
